# Loss of miR-143 and miR-145 in condyloma acuminatum promotes cellular proliferation and inhibits apoptosis by targeting NRAS

**DOI:** 10.1098/rsos.172376

**Published:** 2018-08-29

**Authors:** Xiaoyan Liu, Yu Zhang, Su Wang, Guoying Liu, Liming Ruan

**Affiliations:** Department of Dermatology, the First Affiliated Hospital, Zhejiang University School of Medicine, Hangzhou 310003, People's Republic of China

**Keywords:** miR-143, miR-145, NRAS, proliferation, apoptosis, condyloma acuminatum

## Abstract

The expression profile of miRNAs and their function in condyloma acuminatum (CA) remains unknown. In this study, we aimed to detect the effects of miR-143 and miR-145, the most downregulated in CA samples using high-throughput sequencing, on cell proliferation and apoptosis, to determine a novel therapeutic target for CA recurrence. RT-qPCR was used to validate the lower expression of miR-143 and miR-145 in a larger size of CA samples, and the expression of NRAS in CA samples was significantly higher than self-controls as determined by western blotting assay. Luciferase assay was performed to confirm that miR-143 or miR-145 targeted NRAS directly. Transduction of LV-pre-miR-143 or LV-pre-miR-145 to human papilloma virus (HPV)-infected SiHa cells led to reduced proliferation, greater apoptosis and inhibition of expression of NRAS, PI3 K p110*α* and p-AKT. However, knockout of miR-143 or miR-145 in human epidermal keratinocytes by delivery of CRISPR/CAS9-gRNA for target miRNAs protected cells from apoptosis and upregulated expression of target genes as described above. MiR-143 and miR-145 sensitized cells to nutlin-3a, a p53 activator and MDM2 antagonist, while their loss protected cells from the stress of nutlin-3a. Furthermore, siRNA targeting NRAS showed similar effects on proliferation and apoptosis as miR-143 or miR-145. Taken together, our results suggest that loss of miR-143 or miR-145 in CA protects HPV-infected cells from apoptosis induced by environmental stress, in addition to promoting cellular proliferation and inhibiting apoptosis by targeting NRAS/PI3 K/ATK. Restoration of miR-143 or miR-145 might provide an applicable and novel approach to block the recurrence and progression of CA.

## 1. Introduction

Condyloma acuminatum (CA), most frequently caused by human papilloma virus (HPV) types 6b and 11, is one of the most common sexually transmitted diseases. It is characterized by abnormal hyperproliferation and less apoptosis, which results in high recurrence and necessitates repeated therapy [1]. Therefore, it is important to elucidate the mechanism of CA progression and recurrence, eventually to develop approaches to control this disease.

MicroRNAs (miRNAs), small noncoding RNA molecules of 20–25 nucleotides in length, function in post-translational regulation of gene expression via base-pairing with complementary sequences with mRNA molecules [2]. They have been recognized as important gene regulators during past decades, participating in most investigated biological processes including development [3], organogenesis [4], carcinogenesis [5], apoptosis [6] and cell proliferation [7].

Studies on the relationship between HPV and miRNAs have mainly focused on the expression changes of miRNAs of intraepithelial neoplasia and cervical cancer caused by high-risk HPV and the regulatory mechanism on HPV-mediated carcinogenesis [8–10]. On the one hand, HPV E6 and E7 oncoproteins deregulate the expression of the miR-15/16 cluster, miR-17-92 family, miR-21, miR-23b, miR-34a and miR-106b/93/25 cluster via the E6-p53 and E7-pRb pathways [11–16]. On the other hand, differential cellular miRNAs may influence the expression of papillomavirus genes in a differentiation-dependent manner by targeting viral RNA transcripts [17,18]. Although aberrantly expressed miRNAs in human cervical cancer and head and neck squamous cell carcinoma (HNSCC) are well documented [19,20], the expression profile of miRNAs and the function of candidate miRNAs in CA remain largely unknown.

In the present study, we screened the candidate miRNAs involved in the regulation of CA progression. Then we identified the function of miR-143 and miR-145, the lowest expressed ones in the context of CA, on cellular proliferation and apoptosis. Herein, we report that NRAS was targeted by miR-143 or miR-145 for repression. We showed that repression of NRAS by miR-143 or miR-145 could promote more apoptosis, induced by a p53 activator and MDM2 antagonist, nutlin-3a. Furthermore, inhibition of these target miRNAs promoted cellular proliferation, inhibited apoptosis and showed resistance to nutlin-3a. These results demonstrate that miR-143 and miR-145 are decreased in CA, and they play crucial roles in cellular proliferation and apoptosis in the face of stress signals by targeting NRAS.

## 2. Material and methods

### 2.1. Patients and study approval

Five pairs of HPV6b-positive CA samples and adjacent tissues were used for miRNA microarray assay for candidate miRNA selection. Another 60 cases of CA specimens and 20 cases of HPV-negative skin or foreskin tissues were collected for further validation using quantitative real-time PCR (qRT-PCR). Among these, nine pairs of CA samples and adjacent tissues were used for western blotting assay to detect the expression of target genes. The HPV genotyping was performed using a HPV GenoArray Test Kit (Hybribo, China) following the manufacturer's protocol. The clinical features of 60 cases of CA are listed in table 1.

**Table 1.** Clinical features of 60 cases of CA. Note: this includes one case with diabetes and three cases with kidney transplantation.

clinical features	number
sexuality	
male	33
female	27
age	
< 40 years	41
≥ 40 years	19
genotype of HPV	
HPV 6b	25
HPV 11	24
mixed infection	11
recurrence rate	
incipience	32
recurrence	28
recurrence ≥ 6	10
course of disease	
< 3 months	37
≥ 3 months	23
lesional location	
foreskin	25
anus	17
labia/perineum	18
concomitant disease	
yes	4
no	56

### 2.2. miRNA microarray assay

Total RNA was extracted from HPV6b-positive CA tissues and HPV-negative foreskin tissues using RNAiso™ PLUS (TaKaRa, China) following the manufacturer's protocol. The miRNA microarray assays were conducted by a service provider (LC Sciences, China). In brief, the assays were performed on 5 µg of total RNA samples from each negative control or HPV6b-positive CA specimen. The small RNAs were 3′-extended with a polyadenylate tail, using polyadenylate [poly (A)] polymerase and then ligated to an oligonucleotide tag for later staining with Cy5. Hybridization was performed overnight on a μParaFlo microfluidic chip using a microcirculation pump. After hybridization, images were collected and quantified. The microarray data were submitted to an ArrayExpress database with the accession number A-MEXP-2322.

### 2.3. Cell culture and reagent

Human epidermal keratinocytes (HEKs) (Pansheon Company, China) were maintained for up to five passages with defined keratinocyte serum-free media (Invitrogen, USA). HPV16-positive human cervical carcinoma cell line SiHa and human embryonic kidney cell line 293 T were purchased from the Shanghai Institute of Cell Biology of the Chinese Academy of Sciences (Shanghai, China). The latter two cell lines were routinely cultured in Dulbecco's modified Eagle's medium (Hyclone, USA) with 10% heat-inactivated fetal bovine serum (Invitrogen, USA) and 1% penicillin/streptomycin (Invitrogen). All cells were grown in a humidified (37°C, 5% CO_2_) incubator. Also, miRNA mimics for miR-143 or miR-145, or mimic control, and siRNAs for NRAS or siRNA control, were purchased from RiboBio (China). Nutlin-3a, a p53 activator and MDM2 antagonist, and puromycin were purchased from MedChemExpress Company (China). HEKs or SiHa cells were co-treated with 20 µg ml^−1^ or 40 µg ml^−1^ nutlin-3a for an appropriate time after transfection or transduction.

### 2.4. 3′-UTR Luciferase reporter assays

**Figure 1.**
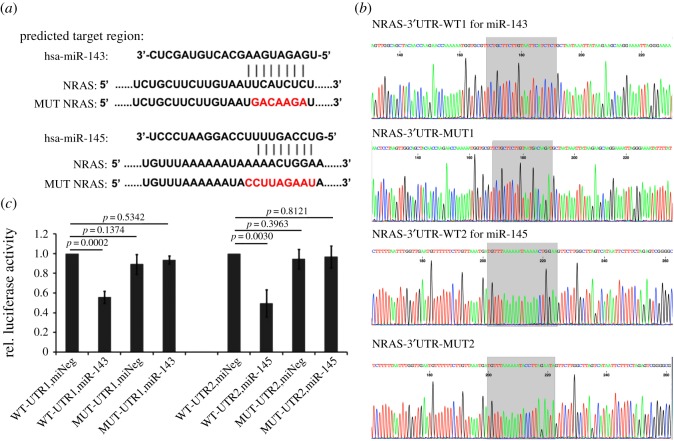
miR-143 and miR-145 target the 3′-UTR of NRAS. (*a*) Sequences show the predicted target site of 3′-UTR of NRAS for miR-143 or miR-145. Mutations to disrupt miR-143 or miR-145 interaction with the 3′-UTR are shown. (*b*) The sequencing results of wild-type or mutant versions of the predicted miR-143 or miR-145 binding sequences in the *NRAS* 3′-UTR. (*c*) Luciferase assays results are shown with the wild- and mutant-type *NRAS* 3′-UTR. Data were mean ± s.d. from at least three experiments.

A wild or mutant NRAS part-length 3′-UTR luciferase reporter construct for miR-143 or miR-145 was made by synthesizing and cloned into the *Xba*I site of GV27 construct (Genechem, China). The target sequences and mutant sites are shown in figure 1*a*. Next, 293 T cells were transfected in 96-well plates by using Lipofectamine 2000 (Invitrogen) with the wild or mutant luciferase reporters (100 ng ml^−1^) and 20 nM miRNA mimics for miR-143 or miR-145, or mimic control. Assays were performed using the dual-luciferase reporter system (Promega, USA) at 48 h post-transfection (hpt). The ratio of the activities of Renilla luciferase over those of firefly luciferase in each well was used as a measure of total reporter activation. The results are averages of data from three independent experiments, assayed in triplicate.

### 2.5. Construction of miRNA overexpressing lentiviral vectors and lentiviral transduction on SiHa cells

The lentiviral vectors overexpressing miR-143 or miR-145 were constructed by the GeneChem Company in China (see electronic supplementary material, figure S1), named LV-pre-miR-143 or LV-pre-miR-145. SiHa cells were sequentially infected with lentiviral particles at a multiplicity of infection (MOI) of 10 in the presence of 5 μg ml^−1^ of polybrene (Sigma, USA). The cells were treated with 0 or 40 µg ml^−1^ of nutlin-3a at 48 hpt for another 24 h, and then harvested for further assays.

### 2.6. Construction of CRISPR/CAS9 plasmids delivering gRNA for miRNAs and their transfection on HEKs

The CRISPR/CAS9 plasmids delivering gRNA for miR-143-3p, miR-143-5p, miR-145-3p or miR-145-5p were constructed by the ViGene Company of China (see electronic supplementary material, figure S2). The double plasmids carrying gRNA-miR143-3p and gRNA-miR-143-5p, or gRNA-miR-145-3p and gRNA-miR-145-5p, were both transfected into HEKs simultaneously using FuGENE HD Transfection Reagent (Promega) according to the manufacturer's protocol. The empty vector was used as a negative control. Cells were treated with 0 or 20 µg ml^−1^ of nutlin-3a and 5 µg ml^−1^ puromycin at 48 hpt, and then harvested for further assays at 72 hpt.

### 2.7. Quantitative real-time PCR

Total RNA was isolated from 60 cases of CA tissues, 20 negative control cases and cells of transfected or transduced groups, using RNAiso™ PLUS (TaKaRa), following the manufacturer's protocol. For mRNA, RNA was subjected to reverse transcription using the reagent kit (TaKaRa), and a Mir-X™ miRNA First-Strand Synthesis Kit (Clontech) was used to produce first-strand cDNA for miRNAs. The amplification reactions were carried out in a 20-μl reaction volume, containing 10 µl of 2 × SYBR^®^ Premix Ex Taq™ and 0.4 µl of 50 × ROX reference dye (TaKaRa). Primers for target mRNAs and miRNAs as well as the internal controls of GAPDH or RNU6 were synthesized by TaKaRa or RiboBio. The *Δ*Ct data were collected automatically. ΔΔCt was calculated by ΔΔCt = average ΔCt of the negative control group –*Δ*Ct of the treated group. The relative expression for a target gene was calculated using 2^−Δ*Δ*Ct^. Each treatment was performed in triplicate, and all experiments were repeated three times.

### 2.8. *In vitro* cellular viability assay, apoptosis assay

SiHa cells or HEK cells, cultured in 96-well plates at a concentration of 4.0 × 10^3^/well or 8.0 × 10^3^/well, were transduced or transfected following the above steps. Then they were treated with or without 40 µg ml^−1^ or 20 µg ml^−1^ of nutlin-3a for one to five days. Cellular viability assay was detected using water-soluble tetrazolium 1 (WST-1) assay at each time point according to the protocol. Each treatment was performed in quadruplicate, and the experiment was repeated three times.

At 72 hpt, the cells in 6-well plates were harvested and resuspended in 100 µl of 1 × Annexin-V binding buffer. The supernatant was incubated with 5 µl of phycoerythrin (PE)-conjugated Annexin V (BD Pharmingen, USA) and 5 µl of 7-amino-actinomycin (7-AAD, BD Pharmingen) for 15 min at room temperature in the dark, and then 400 µl of 1 × buffer was added to each tube. Flow cytometric analysis (BD FACSCalibur, BD Biosciences, USA) was performed within 1 h of staining. Each experiment was performed with at least three biological replicates.

### 2.9. Western blot assay

Total protein was extracted in radioimmunoprecipitation assay (RIPA) buffer (Sigma) containing 1 × Halt Protease and phosphatase inhibitors (Thermo Scientific, USA). For western blotting, equal amounts of total proteins were electrophoresed by SDS-PAGE and then transferred to polyvinylidene difluoride membranes. The antibody for NRAS (ab77392) was purchased from Abcam (USA), and antibodies for AKT (#9272), pAKT-ser473 (#4060), PI3 K-p110*α* (#4249) and PI3 K-p110*β* (#3011) were purchased from Cell Signaling (USA). The members were probed with primary antibodies overnight at 4°C. Following three washes with tris-buffered saline with Tween (TBS/T) buffer, the membranes were incubated with horseradish peroxidase-conjugated anti-rabbit or anti-goat immunoglobulin G (IgG) for 1 h at room temperature. EZ-ECL (Beit-Haemek, Israel) was subsequently used for visualization of the bands. The membranes were stripped and probed with the GAPDH monoclonal antibody (KangChen, China) as the control. Changes in protein levels were measured relative to the internal control GAPDH level, and fold changes were obtained relative to values for the respective negative control (NC). All experiments were repeated at least three times.

### 2.10. Statistics

The results were expressed as mean ± s.d. One-way analysis of variance followed by the Dunnett-t and SNK-q tests was used to assess significant differences among the groups; *p* < 0.05 was considered to be statistically significant.

## 3. Results

### 3.1. Attenuated expression of miR-143 and miR-145 in CA specimens and HPV-infected cells

**Figure 2.**
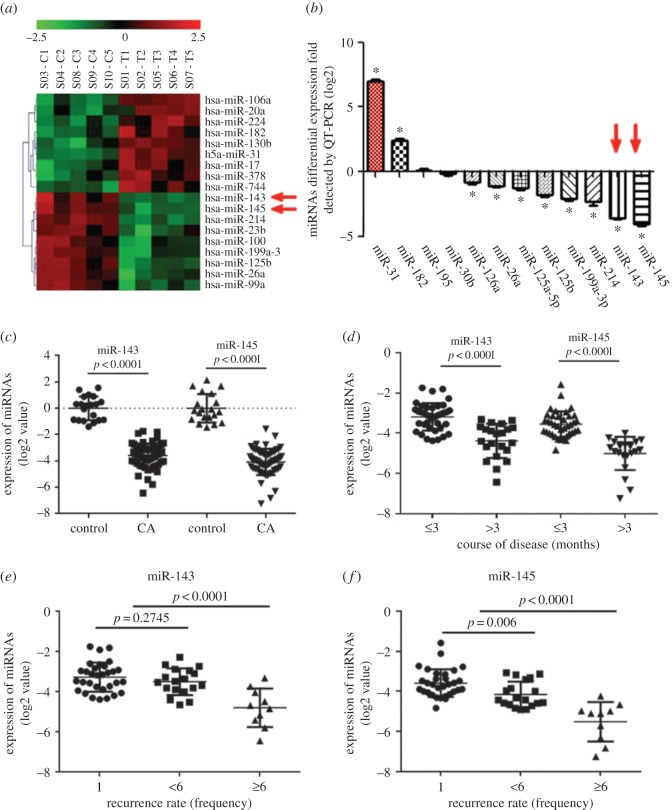
miR-143 and miR-145 expression in CA samples. (*a*) Microarray data of miRNAs in CA samples compared with self-controls (*n* = 5 pairs). (*b*) Expression of candidate miRNAs were detected for CA samples in a larger scale, using qRT-PCR (*n* = 60 in CA samples and *n* = 20 in control group). (*c*) Expression of miR-143 or miR-145 in CA samples using qRT-PCR. Data were expressed as log2 values (*n* = 60 in CA samples and *n* = 20 in control group). (*d–f*) Inverse relationships between expression of miR-143 or miR-145 and course of disease or recurrence rate were revealed.

Differential expression profiling data in the present study are shown in figure 2*a*. Levels of miR-145, miR-143, miR-214, miR-199a-3p and miR-125b were significantly lower in CA specimens than in adjacent tissues (*p* < 0.01), using three-fold expression difference and a positive signal greater than 500 as a cut-off level. In the further validation assay using qRT-PCR, it was confirmed that miR-143 and miR-145 were the two most downregulated miRNAs in the CA group compared with the control group (figure 2*b,c*, *p* < 0.0001). Furthermore, their expression was inversely related to the course of the disease and the recurrence rate (figure 2*d–f*, *p* < 0.0001), whereas no noticeable differences of miR-143 or miR-145 expression were identified between the CA group and the control group in terms of gender, age, genotype of HPV or lesion location (data not shown).

To evaluate the probable effects of miR-143 or miR-145 on CA progression, we detected the expression of candidate miRNAs in SiHa cells and HEKs. As shown in the electronic supplementary material, figure S3A, miR-143 and miR-145 expression was significantly lower in SiHa cells than in HEKs. Therefore, we chose SiHa cells and HEKs as the model cells for further study.

### 3.2. miR-143 and miR-145 directly target the 3′-UTR of NRAS

To elucidate the underlying mechanism by which miR-143 or miR-145 participate in CA progression, we explored their targets by using TargetScan v. 6.2 algorithms [21], miRanda algorithms [22] and miRDB algorithms [23]. To further reduce the false target genes in the analysis, co-predicted target genes by at least two of the three datasets were used for subsequent functional analysis of mRNA targets. Our analysis revealed that NRAS emerged as putative targets of miR-143 or miR-145, based on the predicted results in silico.

**Figure 3.**
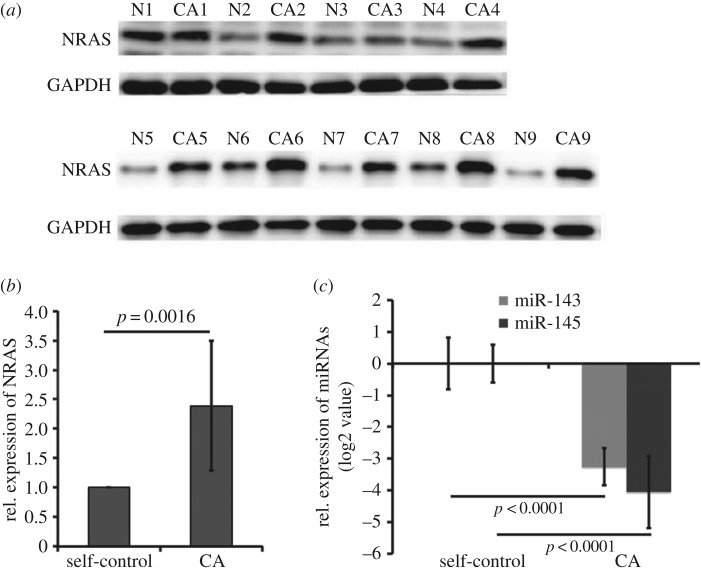
Expression of NRAS in CA samples. (*a*) NRAS protein expression in CA samples and self-controls using western blot assays (*n* = 9 pairs). (*b*) Relative expression of NRAS normalized to self-controls. Data were mean ± s.d. (*n* = 9 pairs). (*c*) Relative expression of miR-143 or miR-145 normalized to self-controls. Data were mean ± s.d. with log2 value (*n* = 9 pairs).

**Figure 4.**
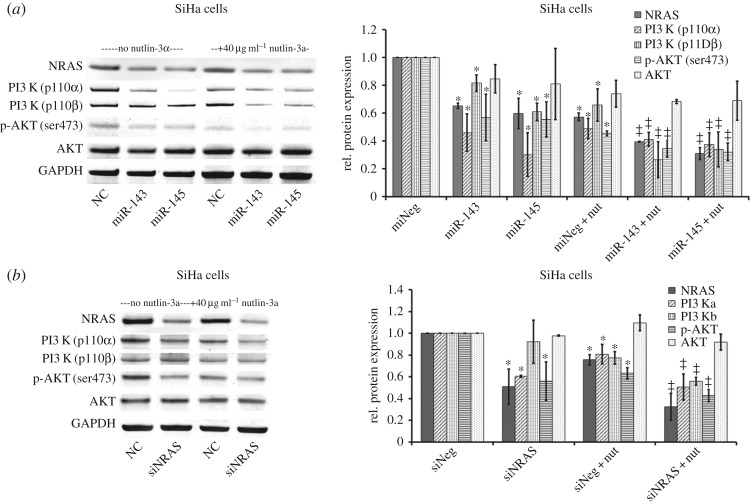
Effect of repression of NRAS on the NRAS/PI3 K/ATK pathway in SiHa cells in response to nutlin-3a. (*a*) SiHa cells were transduced with LV-pre-miRNAs for 48 h and then treated with nutlin-3a for another 24 h. The protein expression of NRAS and target genes in the PI3 K/ATK pathway was analysed by western blotting form at least three separate experiments. (*b*) Assays similar to those performed for panel A, but cells were transfected with siRNAs targeting NRAS (*n* = 3). Graphs from biological replicates are shown on the right. (*p-value of < 0.05 compared with LV-neg or siNeg without nutlin-3a; ^‡^*p*-value of < 0.05 compared with LV-neg or siNeg with nutlin-3a.)

**Figure 5.**
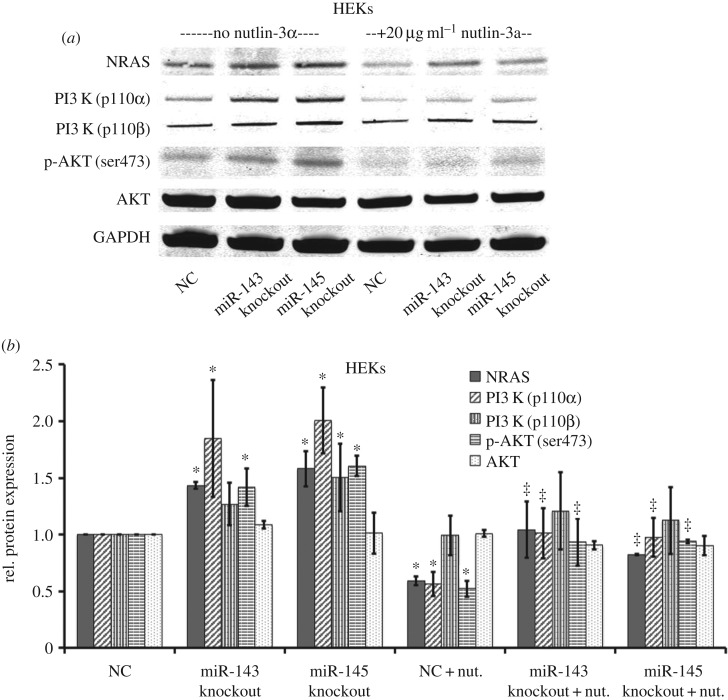
Effect of repression of miR-143 or miR-145 on the NRAS/PI3 K/ATK pathway in HEKs in response to nutlin-3a. (*a*) HEKs were transfected with CRISPR/CAS9-gRNA for miR-143 or miR-145 for 48 h and then treated with nutlin-3a and puromycin for another 24 h. The protein expression of NRAS and target genes in the PI3 K/ATK pathway was analysed by western blotting, and the results from at least three biological replicates are shown in the graph (*b*) on the right. (*p-value of < 0.05 compared with empty plasmid without nutlin-3a; ^‡^*p*-value of < 0.05 compared with empty plasmid with nutlin-3a.)

To confirm the relationships between miR-143 or miR-145 and NRAS, we first examined the protein levels of NRAS in CA and adjacent tissues. The results showed higher levels of NRAS protein with a 2.39 ± 1.10-fold increase in CA samples compared with that in self-controls (figure 3*a,b*, *p* = 0.0016), whereas the expression of miR-143 or miR-145 decreased 3.26 ± 0.58- or 4.05 ± 1.14-fold of log2 values (figure 3*c*, *p* < 0.0001). Furthermore, we also measured protein expression changes of NRAS in SiHa cells transduced with LV-pre-miR-143 or LV-pre-miR-145. We found that miR-143 or miR-145 reduced the expression of NRAS. In addition, we knocked out endogenous miR-143 or miR-145 in HEKs using CRISPR/CAS9 delivery, and observed an increase in protein levels of the target gene (figures 4*a* and 5*a*, *p* < 0.05).

Next, we carried out dual luciferase reporter assay to verify the relationship between miR-143 or miR-145 and NRAS. The sequencing results of wild-type or mutant versions of the predicted miR-143 or miR-145 binding sequences in the *NRAS* 3′-UTR are shown in figure 1*b*. Then they were co-transfected with miR-143 or miR-145 mimics or mimic control into 293 T cells. Luciferase assays were performed at 48 hpt. We found that *NRAS* 3′-UTR luciferase reporter activity was repressed to 0.56 ± 0.06 and 0.49 ± 0.14 by miR-143 and miR-145, respectively (figure 1*c*, *p* = 0.0002 and *p* = 0.0030). We mutated the specific site suspected to be targeted by miR-143 or miR-145 and these mutant 3′UTR reporters were no longer repressed by miR-143 or miR-145 (figure 1*c*). Based on the combined immunoblot data and the *NRAS* 3′-UTR luciferase data, it is suggested that miR-143 or miR-145 targets *NRAS* directly.

### 3.3. Repression of NRAS results in lower expression of the NRAS/PI3 K/AKT pathway in HPV-infective cells in response to nutlin-3a treatment

Previously, it has been shown that the NRAS/PI3 K/AKT pathway usually has an anti-apoptotic role in promoting cellular proliferation, partly due to AKT-dependent stabilization of MDM2 [24]. Inhibition of the PI3 K/AKT pathway sensitized cells to nutlin-3a, an MDM2 antagonist and p53 activator [25]. We hypothesized that miR-143 or miR-145 might repress NRAS expression to inhibit the following cascade signal pathway to play their anti-proliferative and proapoptotic roles. We transduced LV-pre-miR-143 or LV-pre-miR-145 into SiHa cells and measured protein expression changes in NRAS and the following markers of PI3 K p110*α*, PI3 K-p110*β*, p-AKT and AKT. As shown in the electronic supplementary material, figure S3B, the level of miR-143 or miR-145 increased sharply in SiHa cells transduced with LV-pre-miR-143 or LV-pre-miR-145 compared with LV-neg (*p* < 0.0001). We found that miR-143 and miR-145 reduced expression of NRAS, PI3 K p110*α* and p-AKT; however, variable changes of PI3 K-p110*β* and no change of AKT were observed. In addition, we found that nutlin-3a reduced expression of NRAS, PI3 K p110*α* and p-AKT in cells transduced with LV-neg, LV-pre-miR-143 and LV-pre-miR-145, especially in the latter two groups (figure 4*a*, *p* < 0.05). These data suggest that nutlin-3a induces anti-proliferation and apoptosis partly due to inhibition of NRAS/PI3 K/AKT, and inhibition of NRAS/PI3 K/AKT by miR-143 or miR-145 sensitized HPV-infected cells to treatment of nutlin-3a. Similar results were observed using short interfering RNAs (siRNAs) that directly target *NRAS* (figure 4*b*, *p* < 0.05).

### 3.4. Repression of miR-143 or miR-145 results in increased levels of the NRAS/PI3 K/AKT pathway expression in HEKs in response to nutlin-3a treatment

In addition to introducing miR-143 or miR-145 to SiHa cells, we knocked out endogenous miR-143 or miR-145 in primary HEKs using CRISPR/CAS9 delivery. After transfection of double CRISPR/CAS9-gRNAs for miR-143 or miR-145, the expression of miR-143 or miR-145 was underdetermined (electronic supplementary material, figure S3C), while NRAS expression at both transcriptional and protein levels was increased markedly (electronic supplementary material, figure S3D, *p* = 0.0003 and *p* < 0.0001). In HEKs with knockout of miR-143 or miR-145, we observed an increase in protein levels of NRAS, PI3 K p110*α* and p-AKT compared with the negative control. Furthermore, in the presence of knockout of miR-143 or miR-145, nutlin-3a mediated inhibition of NRAS, and following markers were obviously increased (figure 5, *p* < 0.05), suggesting that loss of miR-143 or miR-145 protects cells from the stress of nutlin-3a.

### 3.5. Repression of NRAS plays an anti-proliferative and proapoptotic role in HPV-infective cells in response to nutlin-3a treatment

**Figure 6.**
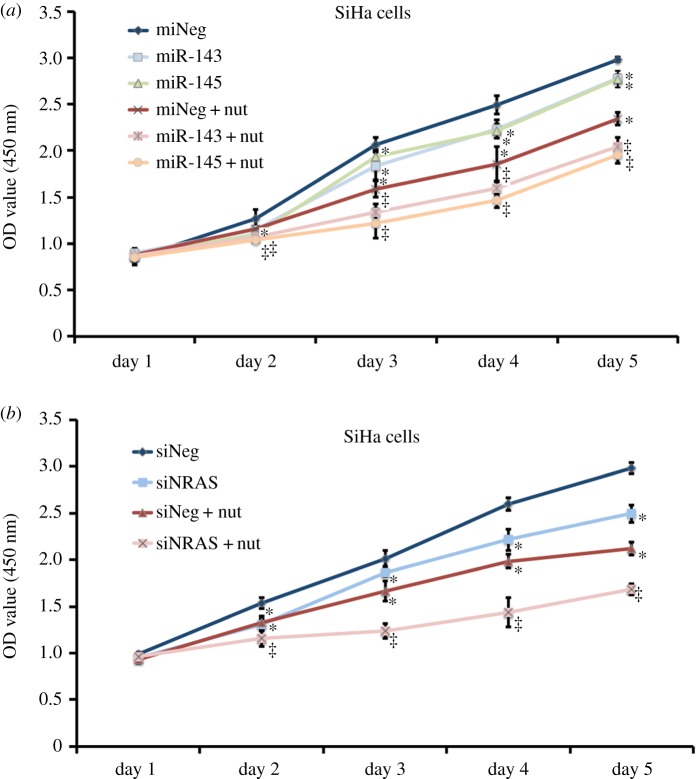
Effect of repression of NRAS on cellular proliferation in SiHa cells in the presence of nutlin-3a. (*a*) Growth curve of SiHa cells transduced with LV-pre-miR-143, LV-pre-miR-145 or LV-neg, treated with 0 or 40 µg ml^−1^ of nutlin-3a for day 1 to day 5. Data were mean ± s.d. using WST-1 assay. (*b*) Assays were similar to those performed for *a*, but the cells were transfected with siRNA targeting NRAS. (*p-value of < 0.05 compared with LV-neg or siNeg without nutlin-3a; ^‡^*p*-value of < 0.05 compared with LV-neg or siNeg with nutlin-3a.)

**Figure 7.**
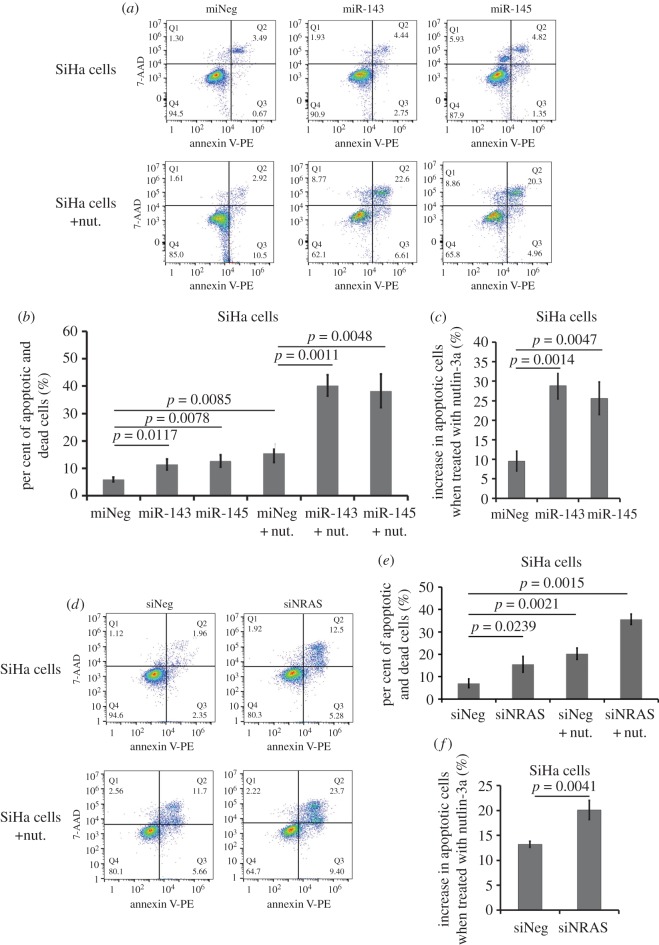
Effect of repression of NRAS on cellular apoptosis in SiHa cells in the presence of nutlin-3a. (*a*) Annexin V-PE/7-AAD assays for apoptosis of SiHa cells transduced with LV-pre-miR-143, LV-pre-miR-145 or LV-neg, treated with 0 or 40 µg ml^−1^ of nutlin-3a. (*b*) Histogram of per cent of apoptotic and dead cells in SiHa cells of each group treated with 0 or 40 µg ml^−1^ of nutlin-3a. Data were mean ± s.d. from at least three experiments. (*c*) Histogram of increase in apoptotic cells of SiHa cells of each group when treated with 40 µg ml^−1^ of nutlin-3a. Data were mean ± s.d. from at least three experiments. (*d–f*) Assays were similar to those performed for (*a*), (*b*) and (*c*), but cells were transfected with siRNA targeting NRAS.

WST-1 assay was performed to investigate the role of miR-143 or miR-145 on cell viability. As shown in figure 6*a*, overexpression of miR-143 or miR-145 in SiHa cells slightly suppressed cell proliferation (*p* < 0.05). We also found that an increase of miR-143 and miR-145 significantly inhibited proliferation of SiHa cells compared with the LV-neg control under the stress of 40 µg ml^−1^ nutlin-3a (*p* < 0.05). We analysed apoptosis using flow cytometry and staining cells with Annexin V-PE and 7-AAD. Overexpression of miR-143 or miR-145 resulted in a slightly increased population of apoptotic cells of SiHa cells (figure 7*a,b*, *p* = 0.0117 and *p* = 0.0078). However, 15.47 ± 3.32% of apoptotic and dead SiHa cells remained in LV-neg group after treatment with 40 µg ml^−1^ nutlin-3a, while in the cells transduced with LV-pre-miR-143 or LV-pre-miR-145, we observed 40.23 ± 3.87% or 38.27 ± 6.12% in the per cent of apoptotic and dead cells (figure 7*a,b*, *p* = 0.0011 and *p* = 0.0048). In addition, we observed a 9.53 ± 2.55% increase in the per cent of apoptotic cells induced by nutlin-3a (figure 7*a–c*, *p* = 0.0085). However, in the cells transduced with LV-pre-miR-143 or LV-pre-miR145, the increase of apoptotic cells obviously rose to 28.67 ± 3.40% or 25.60 ± 4.16% (figure 7*a–c*, *p* = 0.0014 and *p* = 0.0047). Cells transfected with siRNAs to repress NRAS showed a similar sensibility to nutlin-3a-induced anti-proliferation and apoptosis (figures 6*b* and 7*d–f*).

### 3.6. Repression of miR-143 or miR-145 plays a role in promoting cellular proliferation and anti-apoptosis in HEKs in response to nutlin-3a treatment

**Figure 8.**
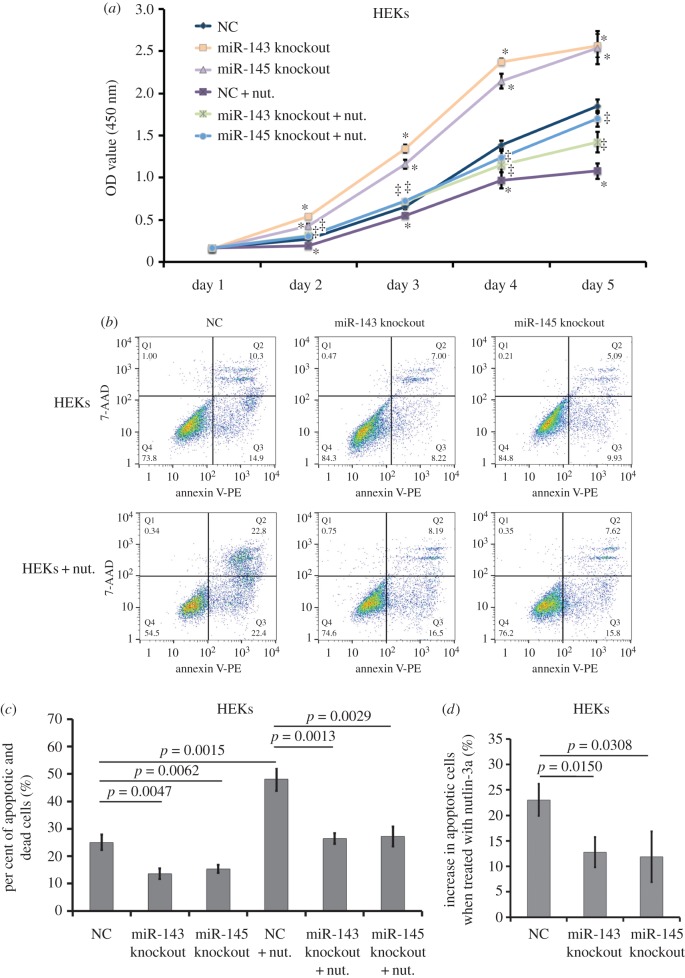
Effects of repression of miR-143 or miR-145 on cellular proliferation and apoptosis in HEKs in the presence of nutlin-3a. (*a*) Growth curve of HEKs transfected with CRISPR/CAS9-gRNA for miR-143 or miR-145 and treated with 0 or 20 µg ml^−1^ of nutlin-3a for day 1 to day 5. Data were mean ± s.d. using WST-1 assay (*p-value of < 0.05 compared with empty plasmid without nutlin-3a; ^‡^*p*-value of < 0.05 compared with empty plasmid with nutlin-3a). (*b*) AnnexinV-PE /7-AAD assays for apoptosis of HEKs transfected with CRISPR/CAS9-gRNA for miR-143 or miR-145 for 48 h and then treated with nutlin-3a and puromycin for 24 h. (*c*) Histogram of per cent of apoptotic cells in HEKs of each group treated with 0 or 20 µg ml^−1^ of nutlin-3a. Data were expressed as mean ± s.d. from at least three experiments. (*d*) Histogram of increase in apoptotic cells in HEKs of each group when treated with 20 µg ml^−1^ of nutlin-3a. Data were expressed as mean ± s.d. from at least three experiments.

Additionally, we sought to determine changes in cellular proliferation and apoptosis in HEKs when miR-143 or miR-145 was knocked out, and found that loss of miR-143 or miR-145 promoted proliferation of HEKs whether or not this was in response to anti-proliferation of 20 µg ml^−1^ nutlin-3a (figure 8*a*, *p* < 0.05). At 72 h after delivery of the CRISPR/CAS9 for miR-143 or miR-145 into HEKs, flow cytometry assays were also performed. The average per cent of apoptotic and dead cells in the control group was 25.07 ± 2.87%, while that in the latter two groups was 13.63 ± 1.96% and 15.33 ± 1.40%, respectively (figure 8*b,c*, *p* = 0.0047 and *p* = 0.0062). Furthermore, when treated with 20 µg ml^−1^ of nutlin-3a, the average per cent of apoptotic and dead cells decreased from 48.10 ± 4.25% in the control HEKs to 26.43 ± 1.97% or 27.20 ± 3.62% in the HEKs with knockout of miR-143 or miR-145 (figure 8*b,c*, *p* = 0.0013 and *p* = 0.0029). Furthermore, in the HEKs with knockout of miR-143 or miR145, the increase of apoptotic cells induced by nutlin-3a markedly dropped from 23.03 ± 3.15% to 12.80 ± 2.98% or 11.87 ± 5.01% (figure 8*b–d*, *p* = 0.0150 and *p* = 0.0308). Collectively, these data provide sufficient experimental evidence that miR-143 and miR-145 perform an anti-proliferative and proapoptotic role in cells that highly expressed miR-143 or miR-145, and sensitize cells to the stress of nutlin-3a.

## 4. Discussion

CA is biologically characterized by abnormal cell proliferation and apoptosis in HPV-infected individuals, seldom undergoing malignant transformation [1].

In previous documents, studies on miRNA signatures related to HPV infection mainly focused on cervical cancer caused by high-risk HPV and reported more variable results [17–20]. As early as 2012, a set of HPV core miRNAs, including the miR-15a/miR-16/miR-195/miR-497 family, miR-143/miR-145 and miR-106-363 clusters, were identified in HPV + HNSCC, which were more similar to HPV + cervical SCC (CSCC) than to HPV - HNSCC [20]. Both high-risk HPV (hrHPV) E6 and E7 oncoproteins can influence expression of cellular miRNAs through their effects on transcription factors, such as p53, c-Myc and E2F [11,26,27]. Recently, HPV16 E6 has been shown to suppress miR-23b expression in CSCC through DNA methylation of the host gene C9orf3 [28]. In addition to the effect of HPV16 E6 on miRNA expression, HPV8 E6 especially downregulates CCTTA/enhancer-binding protein *α* (C/EBP*α*) at the transcriptional level, which can directly bind the miR-203 gene within its hairpin region, and further suppress the miR-203 transcription [29].

With regard to miR-143/145, cervical cancer cells expressed no or very little miR-143/145 clustering; moreover, reduced miR-143/145 expression is common in other tumour types unrelated to HPV infection. They have been reported to act as tumour suppressors, and their overexpression can inhibit tumour growth or invasion, and induce apoptosis, by targeting ERBB3, KRAS, AKT, MDM2 and PAI-1 [30–34]. By contrast, stromal expression of miR-143/145 in lung cancer promotes neoangiogenesis by targeting CAMK1D, an inhibitory kinase [35]. In physiological processes, miR-143/145 promotes smooth muscle cells to endothelial cells to further modulate vessel stabilization [36]; they also are activated independently by Jag-1/Notch signalling to regulate vascular smooth muscle cell differentiation [37]. Furthermore, they regulate odontoblast differentiation and dentin formation through the KLF4 and OSX transcriptional factor signalling pathways [38]. These data imply that the effects of miRNAs occur in a context-specific manner. The effects and their probable mechanism of miR-143/miR-145 on CA progression or recurrence, but not on malignant transformation, remains largely unknown.

In the present study, with five pairs of CA samples caused by low-risk HPV6b and self-nonlesional controls using miRNA microarray assay, we found that the expression of 12 miRNAs was dysregulated in CA, with three showing upregulation and nine showing downregulation. Among these, miR-143 and miR-145 were the two most commonly downregulated miRNAs. These microarray data were validated with larger samples using qRT-PCR. To our knowledge, this study is the first report to analyse the relationships between candidate miRNAs and clinical features of CA and, further, to reveal that reduced miR-143 and miR-145 correlated inversely to the course of disease and recurrence frequencies, although unrelated to gender, age, HPV-type and lesion distribution. Patients with lower levels of miR-143 and miR-145 have a higher recurrence rate and longer disease duration compared with those with higher levels. As discussed, attenuation of miR-143/miR-145 in hrHPV + tissues might be caused by hrHPV E6 or E7 oncoproteins. However, in our study, the expression of miR-143/miR-145 was lower, not only in hrHPV + CA specimens but also in lrHPV + specimens, independent of HPV-types. Further investigation is needed to determine whether miR-143 and miR-145 expression would be suppressed by lrHPV E6 or E7 protein, or other factors.

Gunasekharan & Laimins [39] revealed that both miR-143 and miR-145 were suppressed seven-fold in HPV31 rafts compared with the normal rafts. Furthermore, overexpression of miR-145 in HPV-positive cells resulted in reduced genome amplification to control its own life cycle. In our study, we found that miR-143 and miR-145 were significantly downregulated in HPV16 + SiHa cells, compared with HEKs. By manipulating the levels of candidate miRNAs in SiHa cells by transduction with LV-pre-miRNAs, we proved a slight role of miR-143 and miR-145 in suppressing cellular proliferation and enhancing cellular apoptosis. In addition, when we co-treated the transduced SiHa cells with nutlin-3a to induce cellular apoptosis, an obvious inhibition on cellular proliferation and a significant increase of apoptotic cells between the pre-miRNA group and control group were observed. Furthermore, knockout of miR-143 or miR-145 in keratinocytes showed the opposite effect on proliferation and apoptosis and protected cells from apoptosis induced by nutlin-3a. It is implied that miR-143 or miR-145 might play a role in regulating CA progress or recurrence.

To explore the probable molecular mechanism of these candidate miRNAs in CA pathogenesis, we predicted miRNA targets using the three miRNA datasets and found that *NRAS* emerged as a putative target of miR-143 or miR-145, based on the predicted results in silico.

*NRAS* is a member of the *RAS* oncogene family (which comprises *KRAS*, *HRAS* and *NRAS*). RAS is activated by a complex signal cascade and, in turn, triggers downstream signalling pathways, such as the PI3 K/AKT, MAPK and Ral pathways, associated with uncontrolled cell proliferation and tumour growth [40,41]. In addition to tumorigenesis, reduction of *NRAS* using siRNA or miR-146a reduced the proliferation rate and increased apoptosis in human umbilical vein endothelial cells (HUVECs), resulting in further involvement in peripartum cardiomyopathy [42]. For epidermal development, combined deficiency of *KRAS*, *HRAS* and *NRAS* was associated with epidermal thinning and a dramatic decrease in proliferation of keratinocytes [43]. By contrast, increased expression of *RAS* or expression of oncogenic *RAS* efficiently induced hyperproliferation, papillomas and even SCCs [44]. Our data showed that the expression of NRAS protein in CA samples was higher than that in self-controls. In addition, NRAS in SiHa cells was increased compared with that in HEKs, not only at mRNA levels but also at protein levels. It is suggested that NRAS play a critical role in the progression of CA, especially in forming papillomas. Furthermore, inhibition of NRAS in SiHa cells showed a similar effect on cellular proliferation and apoptosis. Based on the results mentioned above and the luciferase assays between NRAS and miR-143 or miR-145, we infer that in CA, attenuated expression of miR-143 or miR-145 promotes formation of papilloma and inhibition of apoptosis by upregulation of NRAS.

To further investigate the regulatory function of miR-143 or miR-145, we also detected the level of involved proteins in the PI3 K/AKT pathway. Herein, consistent with previous reports, we observed that decreased NRAS by exogenous expression of miR-143, miR-145 or siNRAS in SiHa cells further inhibited the expression of PI3 K p110*α* and p-AKT, whereas loss of miR-143 or miR-145 in HEKs resulted in an enhancement of NRAS followed by an increase of PI3 K p110*α* and p-AKT. The NRAS/PI3 K/AKT pathway usually has an anti-apoptotic role to promote cellular proliferation, partly due to AKT-dependent stabilization of MDM2 [24,45]. Inhibition of the PI3 K/AKT pathway sensitized acute lymphoblastic leukaemia cell lines to nutlin-3a-induced apoptosis [25]. As expected, nutlin-3a inhibited the expression of involved proteins in the NRAS/PI3 K/AKT pathway, sensitized to nutlin-3a, promoted cell proliferation and protected cells from cellular apoptosis induced by nutlin-3a.

In this study, we identified NRAS as a direct target of miR-143 or miR-145 in human keratinocytes and HPV-infected cells. This conclusion is supported by several findings: (i) overexpression of miR-143 or miR-145 in SiHa cells downregulated the expression of NRAS, and attenuated them by inhibitor-restored NRAS expression of HEKs; (ii) a complementary sequence of miR-143 or miR-145 was identified in the 3′-UTR of NRAS mRNA; (iii) overexpression of miR-143 or miR-145 suppressed luciferase reporter activity of the NRAS 3′-UTR-containing vector, and this effect was eradicated by mutation of the miR-143 or miR-145 binding site in the NRAS 3′-UTR; and (iv) inhibition of NRAS showed similar effects on cellular proliferation and apoptosis to those of miR-143 or miR-145.

## 5. Conclusion

In summary, we revealed that miR-143 and miR-145 had an attenuation profile in CA samples compared with normal skin samples. We further demonstrated that miR-143 and miR-145 regulate cell proliferation and apoptosis induced by p53 activator by targeting *NRAS*. Based on our results, lower miR-143/miR-145 may warrant further evaluation for potential clinical applications in CA, indicating a much higher rate of recurrence. Restoration of miR-143 or miR-145 to HPV-infected cells induced apoptosis, which could be applicable to HPV infections in humans with the proper delivery strategy.

## Supplementary Material

Supplementary materials

## Supplementary Material

Figure S1

## Supplementary Material

Figure S2

## Supplementary Material

Figure S3

## Supplementary Material

CRISPR_CAS9-gRNAsequencing

## Supplementary Material

sequencing for LV-Pre-miR145.ab1

## Supplementary Material

sequencing for LV-pre-miR-143.ab1

## Data accessibility

Our data are deposited at Dryad Digital Repository: http://dx.doi.org/10.5061/dryad.407tq [46].
