# Supplementary material for: Loss of miR-143 and miR-145 in condyloma acuminatum promotes cellular proliferation and inhibits apoptosis by targeting NRAS
Source: R Soc Open Sci. 2018 Aug 29;5(8):172376. doi: 10.1098/rsos.172376 (PMC6124073; doi:10.1098/rsos.172376)
Supplement: Supplementary materials [file rsos172376supp1.docx]

**Supplementary materials**

1. Briefly, the pre-miRNA product and the negative control were inserted into the linearized eukaryotic GV254 vector (Figure S1A). All of these vectors were transformed into DH5α chemically competent *Escherichia coli*, and the desired expression clones were identified by PCR amplification and electrophoresis and then were confirmed by sequencing (Invitrogen, Figure S1B). The recombinant pre-miRNA vectors and the packaging vectors (pHelper 1.0 and pHelper 2.0) (GeneChem) were co-transfected into 293 cells with Lipofectamine 2000. The culture supernatants were collected at 48 hpt, concentrated, and used as virus stocks named LV-pre-miR-143 or LV-pre-miR-145. All of the lentiviral vectors expressed enhanced green fluorescent protein (GFP), which allowed for determining of titers and measuring their infection efficiency in infected cells.

2. In brief, the synthesized gRNAs were inserted into the linearized eukaryotic vector pKO-U6-gRNA-CMV-SpCAS9-p2a-Puro (Figure S2A). The following processes of transformation, PCR amplification and electrophoresis were as same to those shown in 2.5, and then the positive plasmids were confirmed by sequencing (Invitrogen, Figure S2B). The effect of CRISPR/cas9-miR-143 and CRISPR/cas9-miR-145 on knockout of miR-143 or miR-145 was shown in Figure S3C and 3D.

Figure S1. Construction of miRNA overexpressing lentiviral vectors. (A) A schematic representation of the GV254 vector. (B) The sequences of LV-pre-miR-143 and LV-pre-miR145 were confirmed by sequencing. (C) GFP-positive SiHa cells in LV-pre-miRNAs-transduced groups at an MOI of 10 (magnification of 100×).

Figure S2. Construction of CRISPR/CAS9 plasmids delivering gRNA for miR-143 or miR-145. (A) A schematic representation of the pSH-U6-gRNA-CMV-SpCas9-P2A-Puro vector. (B) The sequences of miR-143-5p-gRNA, miR-143-3p-gRNA, miR-145-5p-gRNA and miR145-3p-gRNA were confirmed by sequencing.

Figure S3. Expression of target genes in SiHa cells or HEKs. (A) Expression of miR-143, miR-145 and NRAS in SiHa cells or HEKs. The upper bar was the results of NRAS protein using western blotting assay, and the lower bar was the results of miR-143, miR-145 or *NRAS* mRNA using RT-qPCR. (B) Expression of miR-143 or miR-145 in SiHa cells when transduced with LV-neg, LV-miR-143 or LV-pre-miR-145. (C) Amplification curves of miR-143 or miR-145. (D) The expression of NRAS when miR-143 or miR-145 was knocked out. The upper bar was the results of NRAS protein using western blotting assay, and the lower bar was the results of *NRAS* mRNA using RT-qPCR.
