# Supplementary figures and images for: Loss of miR-143 and miR-145 in condyloma acuminatum promotes cellular proliferation and inhibits apoptosis by targeting NRAS
Source: R Soc Open Sci. 2018 Aug 29;5(8):172376. doi: 10.1098/rsos.172376 (PMC6124073; doi:10.1098/rsos.172376)

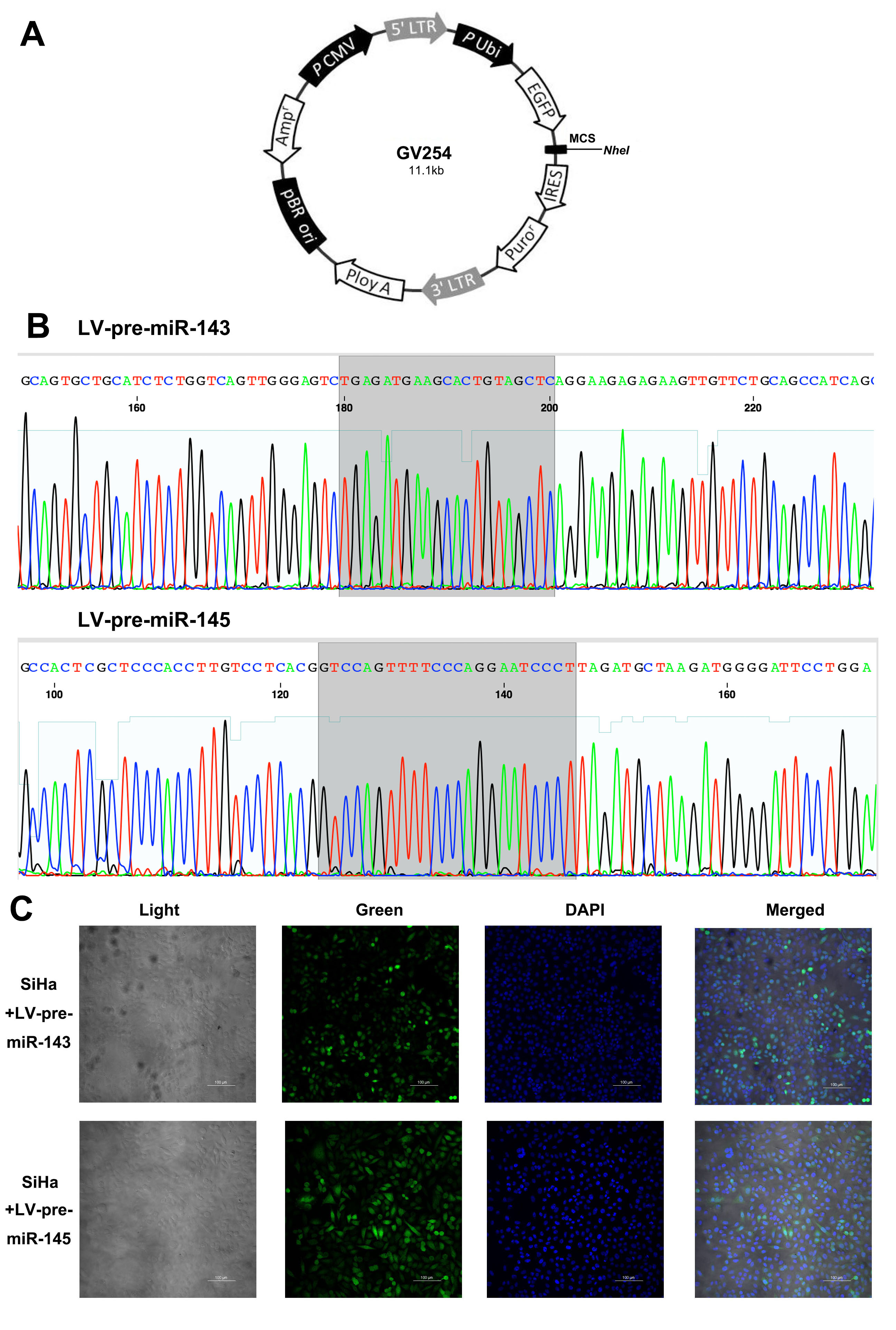

Supplement: Figure S1 [file rsos172376supp2.jpg]

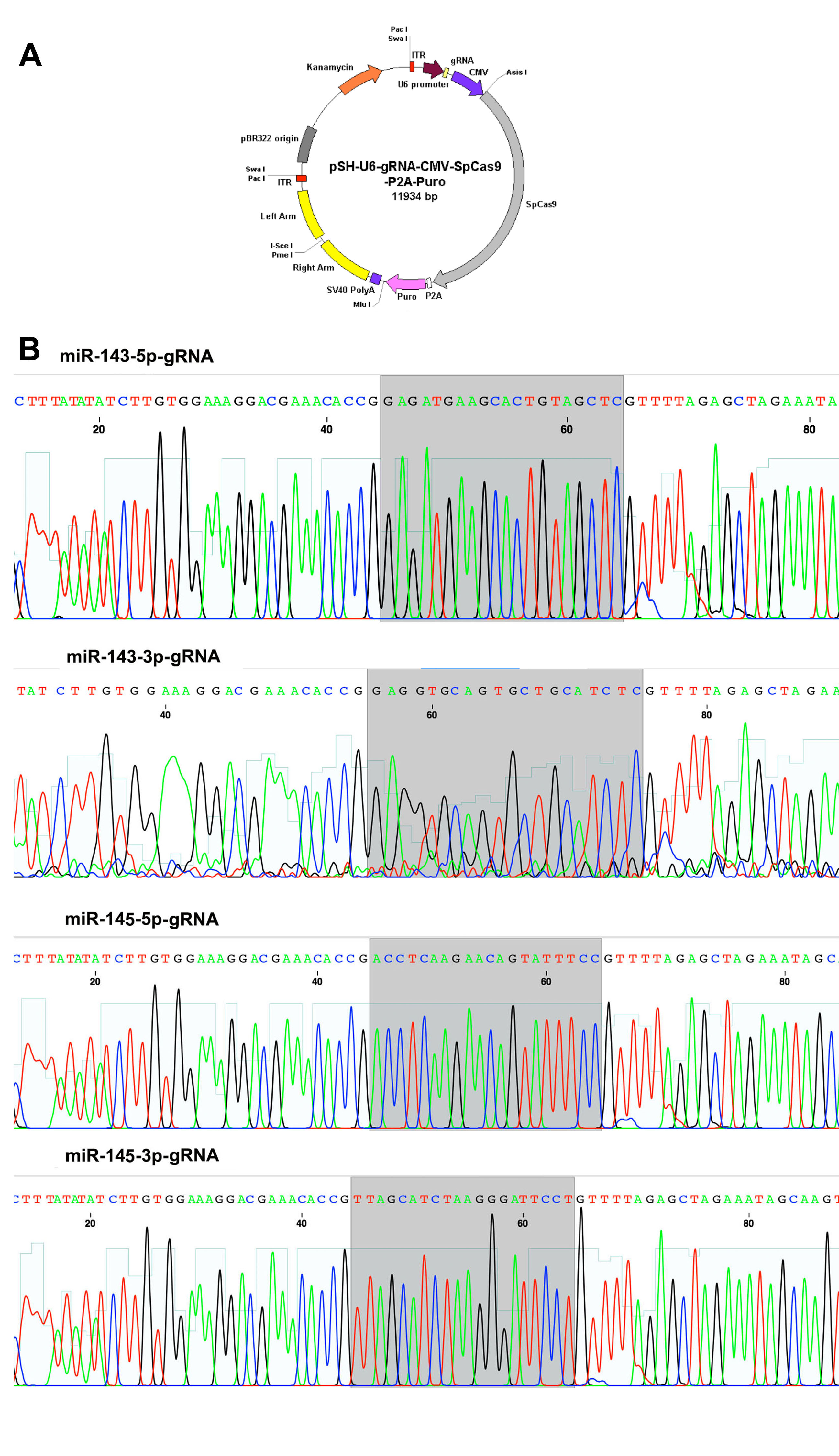

Supplement: Figure S2 [file rsos172376supp3.jpg]

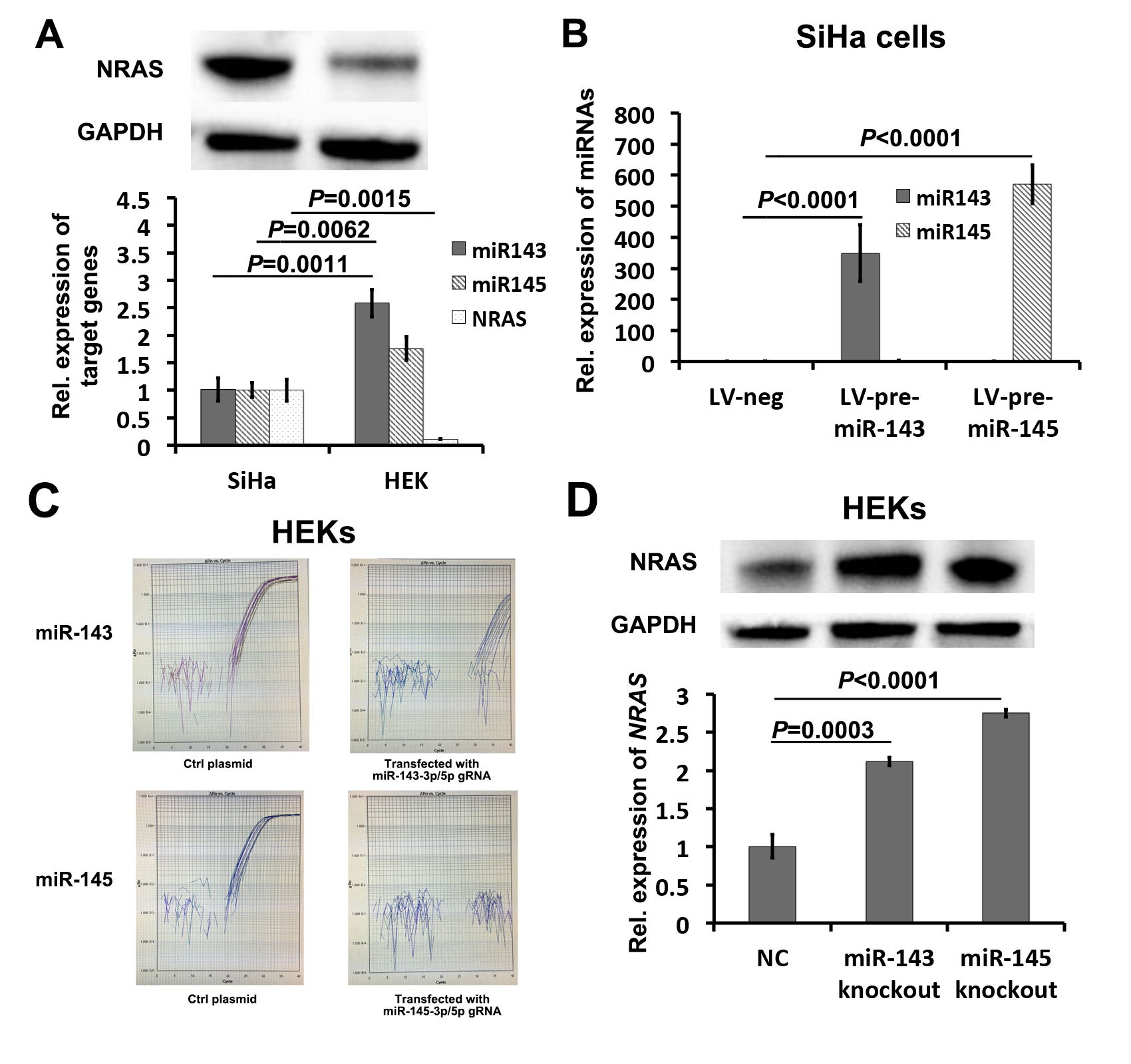

Supplement: Figure S3 [file rsos172376supp4.jpg]
